# Production of Cross-Linked Lipase Crystals at a Preparative Scale

**DOI:** 10.1021/acs.cgd.0c01608

**Published:** 2021-02-17

**Authors:** Raquel Fernández-Penas, Cristóbal Verdugo-Escamilla, Sergio Martínez-Rodríguez, José A. Gavira

**Affiliations:** † Laboratorio de Estudios Cristalográficos, Instituto Andaluz de Ciencias de la Tierra, Consejo Superior de Investigaciones Científicas-Universidad de Granada, Avenida de las Palmeras 4, Armilla, 18100 Granada, Spain; ‡ Departamento de Bioquímica y Biología Molecular III e Inmunología, Universidad de Granada, Avenida de la Investigación 11, 18071 Granada, Spain

## Abstract

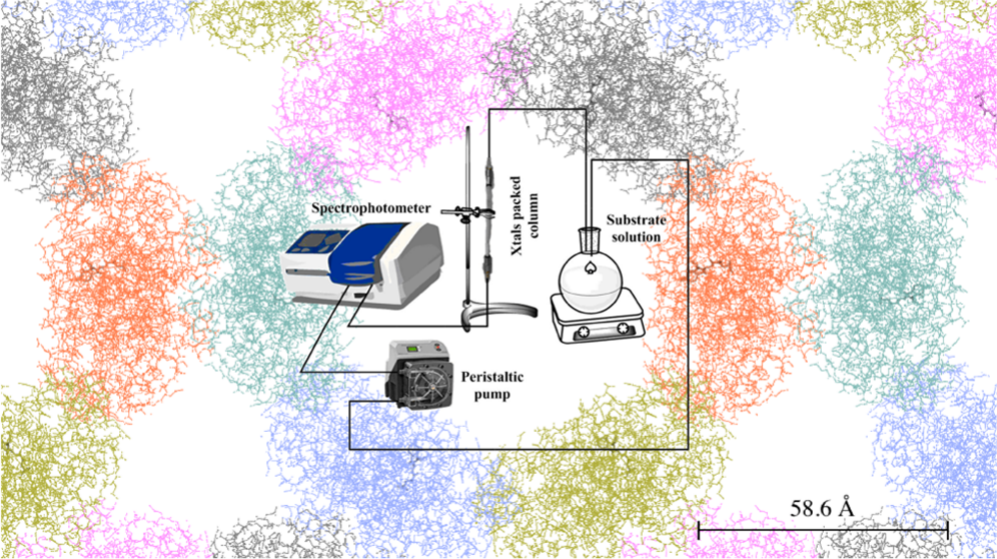

The autoimmobilization of enzymes via cross-linked enzyme crystals (CLECs) has regained interest in recent years, boosted by the extensive knowledge gained in protein crystallization, the decrease of cost and laboriousness of the process, and the development of potential applications. In this work, we present the crystallization and preparative-scale production of reinforced cross-linked lipase crystals (RCLLCs) using a commercial detergent additive as a raw material. Bulk crystallization was carried out in 500 mL of agarose media using the batch technique. Agarose facilitates the homogeneous production of crystals, their cross-linking treatment, and their extraction. RCLLCs were active in an aqueous solution and in hexane, as shown by the hydrolysis of *p*-nitrophenol butyrate and α-methylbenzyl acetate, respectively. RCLLCs presented both high thermal and robust operational stability, allowing the preparation of a packed-bed chromatographic column to work in a continuous flow. Finally, we determined the three-dimensional (3D) models of this commercial lipase crystallized with and without phosphate at 2.0 and 1.7 Å resolutions, respectively.

## Abstract 2

Preparative-scale production (0.5 L) of reinforced cross-linked lipase crystals (RCLLCs) from raw material is facilitated using agarose gel. RCLLCs were active in aqueous solution and in hexane and presented both high thermal and robust operational stability allowing the preparation of a packed-bed chromatographic column to work in continuous flow.

## Introduction

Biocatalysts make use of the versatility, selectivity, and specificity of enzymes to catalyze a variety of processes for the production of relevant compounds under mild conditions,^1^ also allowing the application of continuous flow configurations.^2^ In recent decades, biotransformations have increased to complement classical industrial chemical synthesis processes such as pharmaceuticals, fine chemicals, or foods, facing what has been called the 4th wave of biocatalysis.^3^ Among a wide range of modes to stabilize biocatalysts,^4^ one of the most common strategies to extend enzyme lifetime under extreme conditions and to increase their efficiency is the immobilization in different materials or the autoimmobilization by chemical cross-linking.^5^ Cross-linked enzyme crystals (CLECs) have been described in many different applications, such as biosensing,^6^ drug delivery,^7−10^ biosynthesis,^11^ chromatography,^12^ or material science.^13^ Biotechnologically relevant properties of CLECs,^14,15^ such as their insolubility in aqueous and organic solutions, have been already described for *Candida rugosa* lipase^16^ and more recently for chloroperoxidase from *Caldariomyces fumago*.^17^ CLECs are also mechanically stable^18^ and often present a higher thermal stability^19^ and/or prolonged shelf-life^20^ than the corresponding proteins in a solution. It was initially Vertex Pharmaceuticals (Cambridge, MA) that pioneered the use of CLECs of thermolysin at the industrial level.^21^ Moreover, the use of different CLECs produced on a multikilogram scale was commercialized by Altus Biologics.^22^

Despite the outstanding characteristics of CLECs, such as high operational stability, ease of recycling, or high catalyst and volumetric productivities, the need to crystallize the enzyme has moved the focus to a much easier preparation product known as cross-linked enzyme aggregates (CLEACs), most probably due to the cost and the inherent difficulty of protein crystallization.^23,24^ Still, at the academic level, there is a renaissance in the interest in the CLEC technology, probably boosted by the extensive knowledge gained during the last two decades in protein purification and crystallization, highly decreasing its cost and laboriousness.^20,25−28^ From the pharmaceutical point of view, the most relevant product, already approved by the US FDA, is the TheraCLEC-Total (Anthera Pharmaceuticals), a mixture of CLECs of lipase and protease and CLEAs of amylase, which is administered orally for the treatment of patients with digestive disorders (celiac disease, cystic fibrosis, etc.).^29^

But the real driver of the striking growth in the use of cross-linked protein (enzymes included) crystals (CLPCs) and protein crystals, in general, is the potential application of the regular nanopore materials, with solvent channels ranging from 0.5 to more than 10 nm in diameter, provided by crystallized proteins.^26^ An extreme application is the occlusion of an enzyme within protein crystals such as the entrapment of lipase within the channels of protein crystals for the production of biofuel.^30,31^ Moreover, protein and DNA crystals have also been exploited for scaffold-assisted structure determination.^32^ More recently, it has been shown that it is possible to accurately design large porous assemblies with specific shapes using proteins as building blocks.^33^

As in the case of enzymes, to extend the possibilities of protein crystals, the cross-linking procedure has been introduced to exploit the nanoporosity for a variety of sophisticated and highly functional biomaterials, as containers, vessel reactors, etc.^9^ Several reviews are available summarizing the current strategies to design, guide, and produce protein-based nanomaterials; in all of them, crystal packing is present at all states, from initial characterization to the generation of end products.^32,34−36^ The use of protein crystals as cage scaffolds for the trapping of gases, carry out reaction, or as delivery vehicles, is among the diverse examples of current attempts in the development of these materials. As already mentioned,^30−33^ the tailoring of sequence composition to guide the assembly or the surface porous properties is also a focus of research.^37^ Several research groups have explored the use of protein crystals as biotemplates,^38^ the functionalization of protein crystals with metal ions, complexes, and nanoparticles,^39^ as reaction vessels,^40,41^ trapping different organic molecules including dyes and luminescent compounds,^42,43^ or even for in situ synthesis of luminescent compounds.^44−46^ The entrapment of CdS quantum dots within lysozyme crystals enhances their fluorescence, which can also be modulated.^47^

The use of gels to mimic microgravity conditions (convection-free environment, no sedimentation, etc.) and to improve the diffraction quality of biomacromolecules crystals^48−50^ ends up proving that protein crystals incorporated the gel matrix during their growth, producing new composite materials of improved properties named reinforced protein crystals.^51−55^ The nature of the gel has also been used to modify the properties of CLECs. For example, lysozyme crystals grown in Fmoc-dipeptide hydrogels loaded with single-walled carbon nanotubes were used to produce reinforced cross-linked lysozyme crystals able to conduct electricity.^56^ The entrapment and release of CO using a ruthenium route has also been investigated using lysozyme CLECs^57^ or the empty iron-storage cage protein, apoferritin.^58^ It is also worth noting that from the pharmaceutical application point of view, other cross-linkers such as oxaldehyde (OA) or 1-ethyl-3-(3-dimethylaminopropyl)-carbodiimide (EDC), more favorable to cell viability than glutaraldehyde, have been proposed.^9^

In parallel, there is also an effort to innovate in the production of protein crystals in bulk. Hebel and co-workers showed that *Thermomyces lanuginosus* lipase (TLL) and lysozyme could be crystallized and scaled-up from microliters to 1 L with the help of ionic liquid and with excellent yields of 97 and 95%, respectively.^59^ They also reviewed the most recent uses of crystallization as a purification step. A number of different strategies are being assayed from the combination of a stirred tank with a cooled tubular reactor in bypass,^60^ the force convection evaporation,^61^ continuous flow,^62,63^ or even an airlift,^64^ among several others recently reviewed.^65^

In this work, we demonstrate how crystallization can solve several issues at the industrial level for the use of protein crystals. First, TLL is purified from a commercially available detergent additive following a sequential rough precipitation/crystallization step. Crystallization is then improved using different techniques and final conditions are adapted to the most easily scalable batch in the gel crystallization technique. The advantage of using a gel matrix media, e.g., agarose, is then exploited to: (i) produce homogeneous batches of crystals (500 mL), (ii) facilitate crystal stabilization by chemical cross-linking, and (iii) to obtain high-quality crystals (allowing to determine the three-dimensional (3D) model at 1.7 Å) and to facilitate crystal recovery. Reinforced cross-linked lipase crystals (RCLLCs) can then be lyophilized and stored. The retention of enzymatic activity is shown and used to produce a chromatographic packed column to work in a continuous flow. We demonstrate that, contrary to belief,^66^ the production of reinforced cross-linked enzyme crystals (RCLECs) is a cost-effective way to obtain highly stable enzymatic materials.

## Materials and Methods

### Protein and Chemicals

Reagents were purchased from Sigma-Aldrich and agarose (D-5) was provided by Hispanagar. Capillaries, crystallization kits, and Granada crystallization boxes (GCBs) were purchased from Triana Science & Technology (Granada, Spain). An Aspergillus sp.-stabilized lipase (triacylglycerol acylhydrolase, EC 3.1.1.3.) solution was purchased from Biocon (Barcelona, Spain; Cod. No. 10256, density of 1.21 g·mL^–1^) as Biolipase L (BioL from now on) containing 1% (w/w) enzyme (personal communication). Extensive dialysis of the commercial BioL solution was carried out using MilliQ water in a ratio 1:1000 at 4 °C. Dialysis against other buffers such as Tris–HCl or *N*-(2-hydroxyethyl)piperazine-*N*′-ethanesulfonic acid (Hepes) was also assayed.

For crystallization, the BioL sample was concentrated to 30 mg·mL^–1^ using a theoretical value for the extinction coefficient at 280 nm of 1.2 mL·mg^–1^·cm^–1^ and filtered through a 0.45 μm pore-size filter membrane system (Millipore).

### Protein Crystallization and Cross-Linking Experiments

**Figure 1:**
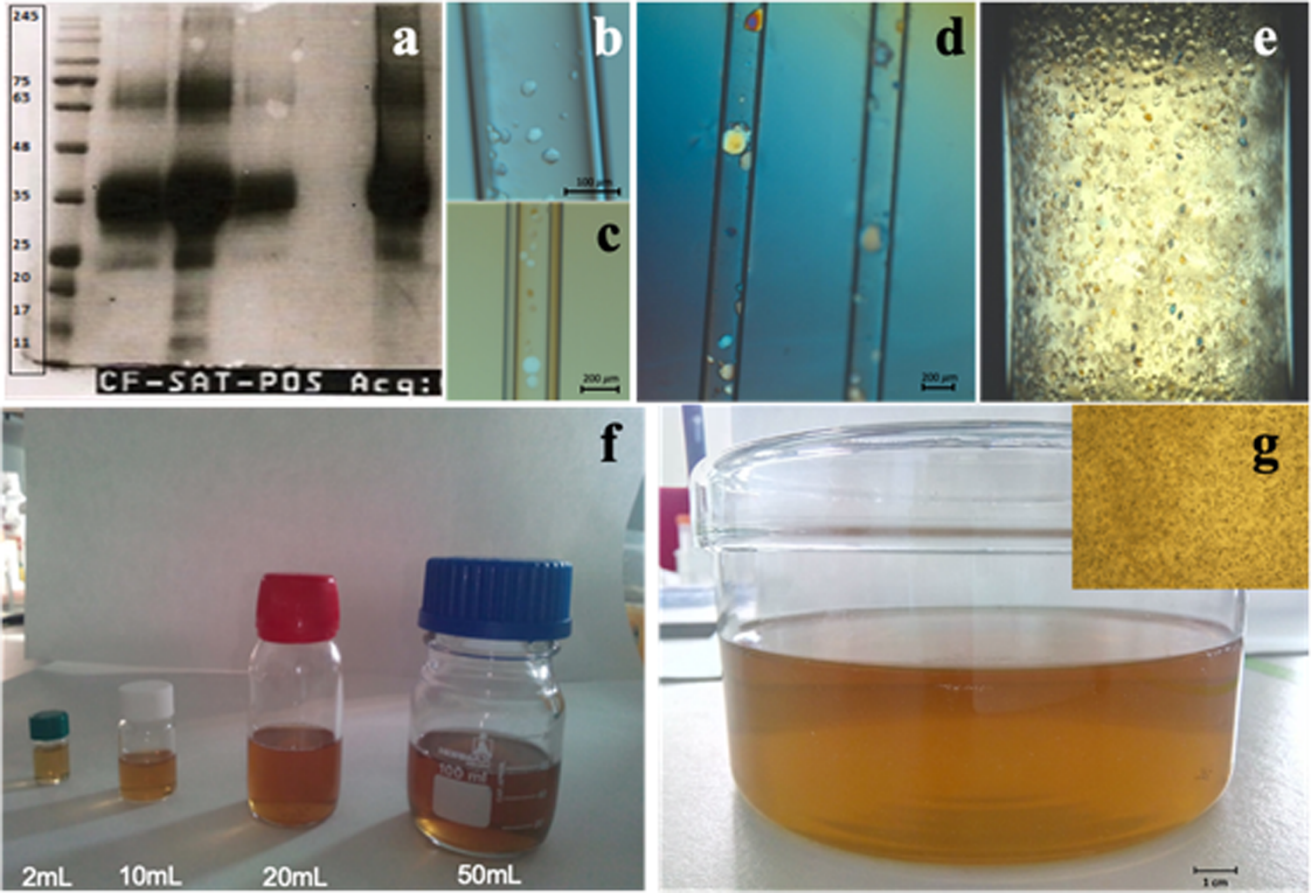
Crystallization protocol from initial screening of BioL to the sequential scale up to 0.5 L. (a) Sodium dodecyl sulfate-polyacrylamide gel electrophoresis (SDS-PAGE) gel of BioL along the purification steps: lane 1, the protein marker; lane 2, after dialysis; lane 3, concentrated; lanes 4 and 5, the dissolved pellet and effluent of the precipitated sample; and lane 6, the commercial product. Crystallization hits found with conditions CSK-23 (b) and sodium formate pH 7.0 (SF7) (c). (d) Crystallization in the presence of 2.5% v/v TMOS (left capillary) and 0.1% w/v agarose (right capillary) using CSK-23 as a precipitating agent. (e) Batch crystallization experiment using agarose 0.1% w/v. (f) Scale-up process is illustrated going from 2 to 50 mL and (g) the last step growing-up to half liter in which the top-right inset corresponds to a closer look to show the homogeneity of lipase crystal size. The agarose concentration from 2 to 500 mL was set at 0.2% w/v.

An initial test of purification through crystallization was attempted using ammonium sulfate at different concentrations mixed directly with the commercial BioL but since the different lot presented different characteristics, i.e., different colors from transparent yellow to dark brownish, we decided to start by cleaning the initial solution by dialysis as much as possible. The commercial lipase was dialyzed against MilliQ water and Tris–HCl (100 mM, pH 7.0), as a final step, and concentrated to approximately 30 mg·mL^–1^ for the screening of crystallization conditions (Figure 1a). We employed the counterdiffusion technique with different commercial crystallization kits (GCB-CSK, Mix-PEG, ammonium sulfate, sodium formate), with a setup consisting of the protein solution loaded into capillaries with a 0.2/0.3 mm inner diameter.^67^ Several conditions produced crystalline material, but the best-looking crystals were obtained with condition #23 (0.82 M K/NaH_2_PO_4_, 0.1 M Hepes pH 7.5) of the CSK and SF7 (5.0 M sodium formate, 0.1 M Tris–HCl, pH 7.0) as precipitants (Figure 1b,c). To assess the effect of agarose and silica gels in crystallization, the BioL solution was mixed with either agarose or tetramethyl orthosilicate (TMOS) to reach a final concentration of 0.2% (w/v) and 2.0% (v/v), respectively, using the two previously identified crystallization conditions (Figure 1d). Based on the results and simple handling, agarose and condition #23 were used to optimize protein and precipitant concentrations, keeping agarose concentration at 0.1% (w/v) (Figures S1 and S2). After fixing precipitant and protein concentrations, we evaluated the influence of agarose over the nucleation density (Figure S3). The selected conditions (30 mg·mL^–1^, 0.82 M K/NaH_2_PO_4_, 0.1 M Hepes pH 7.5, 0.2% w/v agarose) were then subjected to a scale-up process going from 100 μL to 500 mL sequentially (Figure 1e,g).

All of the experiments were kept vertically (counterdiffusion) and stored at 20 °C in an incubator. BioL crystals obtained in capillaries and in gelled batch, with 0.2% (w/v) agarose, were tested for X-ray diffraction.

**Figure 2:**
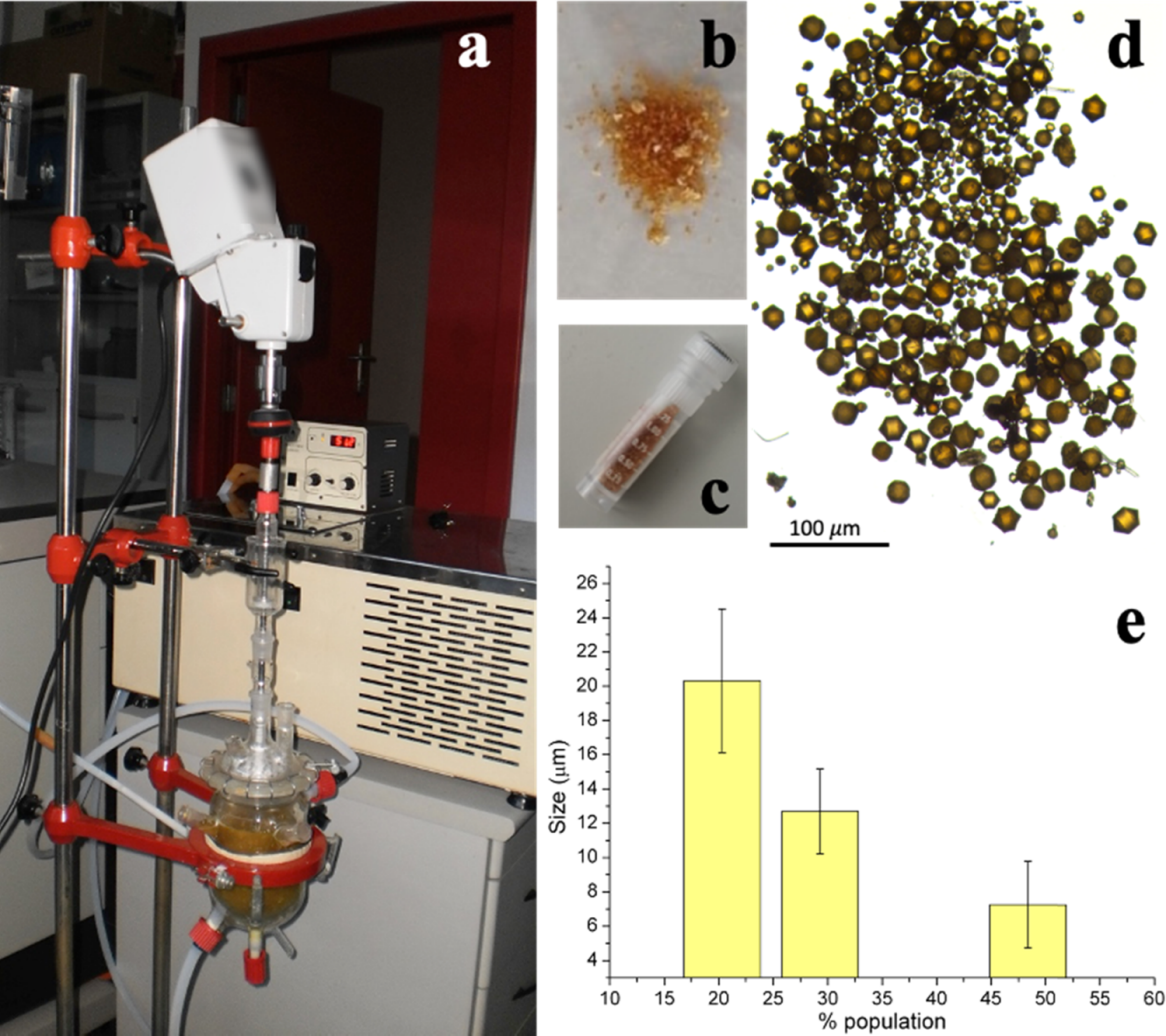
(a) Adaptation of a crystallization instrument to extract the RCLLCs from the agarose matrix. (b) Lyophilized RCLLC final product collected in a 2 mL vial (c). (d) Magnification of the collected RCLLCs and (e) size distribution grouped in three categories to sum up the 100% of crystal population.

Crystal cross-linking was carried out by addition of glutaraldehyde to a final concentration of 2.5% v/v prepared in 0.1 M K/NaH_2_PO_4_ 0.1 M Tris–HCl pH 7.0. After 48 h, crystals were cleaned to remove the agarose gel. The cleaning protocol was tested manually at a small scale and applied to the experiment at a preparative scale. After removing the cross-linker solution, the gel containing the RCLLCs was poured into a glass bulk-crystallization reactor containing 500 mL of water under continuous vertical agitation and kept at 50 °C (Figure 2a). Water was replaced twice and finally removed to recover the crystals and the remaining agarose gel. Finally, the wet mix was lyophilized (lyophilizer Telstar-LyoQuest, −55 °C/ECO) for 24 h to obtain the dry product (RCLLCs) used in all enzymatic assays (Figure 2b–d). Crystal-size distribution was determined from the images of the lyophilized sample using ImageJ,^68^ as shown in Figure S4.

### Data Collection and Refinement

A small volume of the gel containing the crystals, approximately 5 μL, was transferred to a plastic Petri dish with the help of a 100 μL micropipette using a cut tip. The selected BioL crystals were fished out of the gel drop with a loop and transferred to a 5 μL drop of the mother solution containing either 15 or 20% (v/v) glycerol as a cryoprotectant. Crystals grown in capillaries were extracted from the capillary and cryoprotected as already described elsewhere.^69^ After soaking for less than 30 s, crystals were flash-cooled in liquid nitrogen and stored. X-ray diffraction data collection was carried out at XALOC beamline of the Spanish synchrotron radiation source (ALBA) and at ID30A-3 and ID30B-1, beamlines of the European synchrotron radiation source (ESRF). Reflections were recorded on Pilatus detectors and data were indexed and integrated using XDS^70^ and scaled with Aimless^71^ from the CCP4 suite.^72^ The crystal structure of BioL was determined by the molecular replacement method with Molrep^73^ using the structure of *T. lanuginosus* lipase (TLL) as an input model determined at 2.5 Å (PDB ID: 6HW1) after removing any heteroatoms and water molecules. Refinement was done with PHENIX^74^ and completed with Refmac,^75^ with cycles of manual rebuilding using COOT.^76^ The final refined models were checked with Molprobity.^77^ Data collection and refinement statistics are summarized in Table 1. Coordinates and structure factors have been deposited at the PDB with IDs 7APN and 7APP for the models were determined from phosphate and formate precipitant agents, respectively.

**Table 1:** Data Collection and Refinement Statistics (Values in Parentheses are for the Highest-Resolution Shell)

PDB ID	7APN	7APP
space group	*P*6_1_	*P*6_1_
unit cell		
*a*, *b*, *c* (Å)	141.187, 141.187, 80.234	137.851, 137.851, 79.943
ASU	2	2
resolution (Å)^a^	67.08–2.0 (2.07–2.00)	34.46–1.70 (1.76–1.70)
*R*_merge_ (%)^a^	14.97 (95.75)	73.31 (166.8)
*I*/σ*_I_*^a^	8.90 (1.77)	50.68 (3.30)
completeness (%)^a^	99.94 (99.98)	99.97 (100.00)
unique reflections^a^	61 367 (6100)	94 292 (9365)
multiplicity^a^	6.9 (7.3)	42.5 (39.2)
Wilson *B*-factor	29.08	19.325
CC (1/2)^a^	0.991 (0.659)	0.868 (0.846)
refinement		
*R*_work_/*R*_free_ (%)	21.29/25.04	12.70/15.45
no. atoms	4934	5216
protein	4460	4660
ligands	58	76
solvent	416	480
*B*-factor (Å^2^)	36.76	25.95
RMS deviations		
bond lengths (Å)	0.015	0.019
bond angles (deg)	1.90	2.19
Ramachandran (%)		
favored	96.44	95.88
outliers	0.00	0.19

a Statistics for the highest-resolution shell are shown in parentheses.

### RCCLC Activity Assay

The hydrolytic activity of RCLLCs on using 4-nitrophenyl butyrate (*p*NPB) was investigated. A *p*NPB stock solution of 50 mM in anhydrous acetonitrile was prepared and stored at 4 °C until use. The activity was measured using 100 mM NaH_2_PO_4_, 150 mM NaCl, 0.5% Triton-100X pH 7.2 (reaction buffer, RB), using in all cases a concentration of AcN lower than 1%. *p*-Nitrophenol (*p*NP) formation was determined at 400 nm using a Cary 1E UV–VIS spectrophotometer (Agilent Technologies). The concentration of *p*NP produced was determined from a calibration curve (Figure S5). Spontaneous hydrolysis of *p*NPB was subtracted in all measurements using a blank.

RCLLCs (0.5 mg) were added to 20 mL of 400 μM *p*NPB in RB and incubated at 25 °C for 15 min using a thermoshaker (Lan Technics, 150 rpm). Samples were then centrifuged and removed carefully with a micropipette to minimize the loss of enzyme crystals. The absorbance of the supernatant was measured at 400 nm. The activity of the enzyme in a solution was also determined using the same final volumes described for the RCLLC experiments but using a BioL concentration of 50 μg·mL^–1^.

Reuse of RCLLCs was evaluated using 1.0 mg of RCLLCs in 20 mL of 400 μM *p*NPB in RB and incubating at 25 °C for 15 min using a thermoshaker (Lan Technics, 150 rpm). The supernatant was removed carefully and the crystals were washed with 1 mL of water. Crystals were then centrifuged, the aqueous solution was removed, and 20 mL of 400 μM *p*NPB in RB was added to repeat the reaction cycle.

The enzymatic activity of RCLLCs and free BioL was also assayed using heptane as a solvent and α-methylbenzyl acetate (αMA) as a substrate. αMA (65 μL) was added in 500 μL of heptane (final substrate concentration 0.8M). Then, 5 mg of prehydrated RCLLCs suspended in 30 μL of water or 15 μL of a BioL solution (30 mg·mL^–1^) was added. Samples were incubated for 24 h at 40, 50, and 60 °C, with continuous shaking (150 rpm). Thin-layer chromatography (TLC) was used to qualitatively analyze the reaction product developed using a mixture of heptane and ethyl acetate, in a ratio 4:1, as the running solvent. Both the substrate and the product of the reaction (1-phenylethanol) at two concentrations (pure and 1/10 dilution) were included in the TLC plates as references.

### Preparative-Scale BioL-Column Preparation

**Figure 3:**
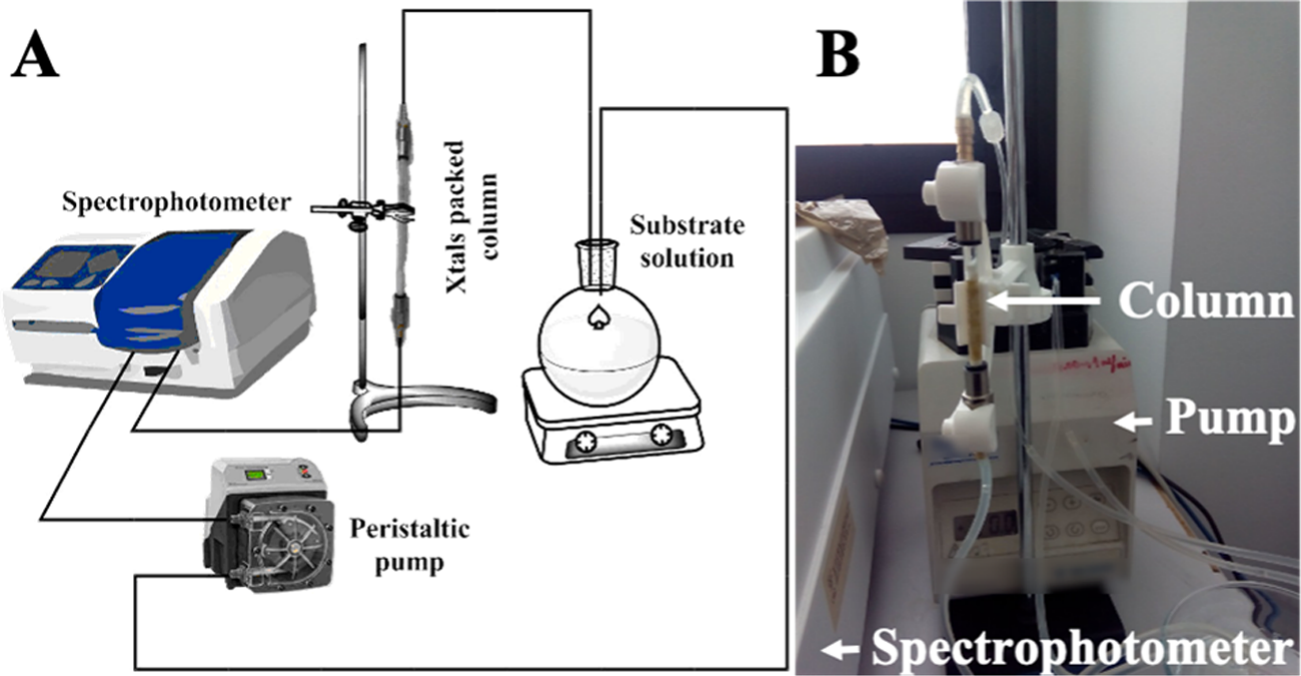
Operational setup of the RCLLC packed-bed column connected to a spectrophotometer and a pump. The system is schematically represented in (A), whereas (B) shows a picture of the column connected to the pump.

RCLLCs (60 mg) were mixed with 1 g of glass beads (SIGMA, G1277, Glass beads, acid-washed, 212–300 μm) and packed in a handmade 10 cm chromatographic column (4.0 mm inner diameter glass cylinder), using glass wool at the top and the bottom of the column to support the stationary phase (Figure 3A). The column was connected to a peristaltic pump (Gilson, Minipuls 3), and pNPB solutions (50–200 μM in RB) were pumped at 0.25–1.0 mL·min^–1^. Conversion to *p*NP was monitored continuously at 400 nm by connecting the tubing to a CARY 100 spectrophotometer (Agilent Technologies) equipped with a thermostatic element. Spontaneous hydrolysis of *p*NPB was also continuously measured and subtracted from enzymatic activity. The whole setup is shown and schematized in Figure 3.

## Results and Discussion

As a proof of concept, we selected lipases to carry out our research for two main reasons. First, lipases are very versatile enzymes that catalyze the hydrolysis of ester linkages, primarily in neutral lipids such as triglycerides, which have been extensively characterized and used as CLECs in many studies and from different sources representing an excellent example of a biocatalyst and its applications.^78,79^ Second, lipase is available as an industrial detergent additive at a reasonable price.

Although the commercial lipase solution was not totally pure (Figure 1a), BioL crystallization was achieved by directly using the crude commercial material after dialysis with MilliQ water with no further purification steps. Initial hits were obtained by the capillary counterdiffusion technique using 0.82 M K/NaH_2_PO_4_, 0.1 M Hepes, pH 7.5 (C23 of the CSK screening kit) or 5.0 M sodium formate in 100 mM AcNa pH 7.0 as precipitating agents (Figure 1). In a second step, we tested the compatibility of both conditions with the use of agarose (0.2% w/v) or silica (2.0% v/v) gels by loading the capillary with the mix of BioL and gels. Although crystals were obtained with both gels, agarose was selected for further development not only due to its ease of handling and the low concentration required to maintain a convection-free media but also because it avoids crystal sedimentation and simplifies the crystal’s extraction procedure (see below). Moreover, silica gels tend to interact strongly with the precipitant, in some cases, provoking the flocculation of the mixture.^80^

Protein and precipitant concentrations were optimized using the batch method while keeping the agarose concentration at 0.1% (w/v). When the nucleation density was too low at 50 mM K/NaH_2_PO_4_, the amount of the precipitate increased strongly at 150 mM (Figure S1c). By fixing the amount of the precipitant at 100 mM, we determined that 15 mg·mL^–1^ was the best protein concentration (Figure S2) and used this condition to study the influence of the agarose concentration. As in the case of lysozyme, insulin, and proteinase K,^81^ increasing agarose concentration also induces the nucleation of BioL (Figure S3). We did not explore this effect further but assayed the low-gel-concentration regime, 0.05–0.3% w/v, with small volume configurations and fixed at 0.2% w/v at volumes greater than 2.0 mL (Figure 1e,f). We determined that the minimum amount of agarose that was able to maintain the homogeneity and integrity of the crystallization media was 0.2% w/v, which is in agreement with the reported rheological characterization.^82^ Below this concentration, the media could collapse when using volumes larger than 2.0 mL. Therefore, the final optimized batch experiments consisted of 500 mL of a protein solution (final concentration of BioL 15 mg·mL^–1^) in 0.1 M K/NaH_2_PO_4_, 0.1 M Tris pH 7.0, and 0.2% (w/v) agarose (Figure 1g). Using this condition, we obtained crystals of size ranging from 8 to 20 μm, with 8 ± 2.52 μm being the most abundant at almost 50% of the sample (Figure 2e).

We used BioL crystals obtained in 0.2% (w/v) agarose to determine the 3D structural model by molecular replacement and using our previous TLL structure as a search model determined at 2.5 Å at room temperature from crystals grown in microchips (PDB ID 6WH1).^83^ The best BioL crystals diffracted X-ray to a resolution of 2.02 Å, slightly better than the source of phase model 6WH1. The crystals obtained belong to the same hexagonal space group (*P*6_1_), with two monomers in the asymmetric unit and superimposed over the dimer of 6WH1 with a root-mean-square deviation (RMSD) value of 0.43 Å. Both structures present the glycosylation of asparagine 33 with an N-acetyl glucosamine (NAG) moiety and phosphate ions as a part of the crystal contacts. Interestingly, the only common phosphate-ion moiety among both models did not match their position but appears 2.9 Å apart. For comparison purposes, we also determined the 3D structure of BioL obtained in the absence of phosphate from crystals grown in capillaries with sodium formate as a precipitant agent. Interestingly, these crystals also belong to the same space group, *P*6_1_, but diffracted X-ray to a resolution of 1.7 Å (Table 1), being the highest-resolution structures of TLL determined in the hexagonal space group and deposited in the PDB.^84^ Besides the great improvement on the resolution, when compared with the structure obtained using phosphate, chains A superimposed with an RMSD value of 0.34 and 0.49 Å when superimposing A and B chains at the same time using STAMP.^85^ Therefore, any given details are referred to the 3D model obtained with phosphate (PDB ID 7APN).

**Figure 4:**
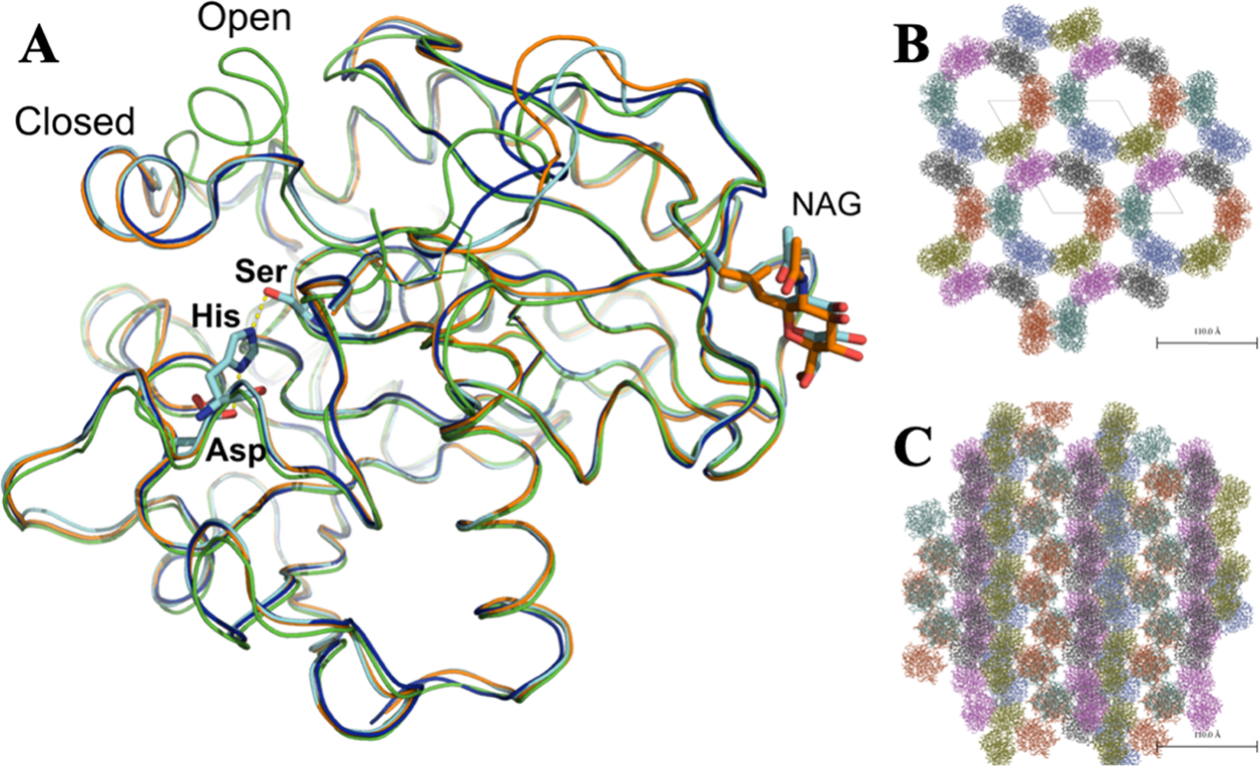
(A) Superposition of the lipase structural models showing the catalytic triad, Ser–His–Asp, of BioL and the NAG moiety at residue Asn33. BioL models in cyan and orange correspond to this work, PDB IDs 7APN and 7APP, respectively. The closed and open forms of TLL corresponding to the 1DT3 and 1EIN PDB models are shown in blue and green colors, respectively. (B, C) Crystal packing.

In short, the structure of BioL corresponds to the typical α/β fold highly preserved among different species and has no difference in the catalytic triad composed by Ser146–His258–Asp201 with the nucleophilic Ser146 (Figure 4), showing the strained conformation characteristic of the nucleophilic elbow.^86^ The structural model presents the closed state of the lid (residue 86–92), as present in many other structural models,^87^ which does not prevent the enzymatic activity in the crystals form as shown next. The crystal packing is shown in Figure 4B,C, in which the main channel of more than 60 Å facilitates the substrate diffusion in the crystals.

### Characterization of RCLLCs

BioL agarose-grown crystals were cross-linked with glutaraldehyde and extracted from the gel following the procedure described above. We obtained 337 mg of dry RCLLCs from 0.5 L of bulk crystallization, resulting in approximately 14.1% yield, estimated from the initial BioL concentration, measured spectrophotometrically at 280 nm. RCLLCs were used to carry out a succinct enzymatic characterization and to prepare the 10 cm chromatographic column for the semipreparative-scale experiments.

Although BioL was crystallized in its closed-lid form, the enzyme was active in water and organic media. It has already been discussed and demonstrated that the lid movement of TLL is not necessary at all for the enzymatic reactions, although it may play a relevant role for the substrate enantioselectivity and produce a diminution in its activity,^88^ although still today the open/closed conformations are wrongly linked to the active/inactive forms.^89^

**Figure 5:**
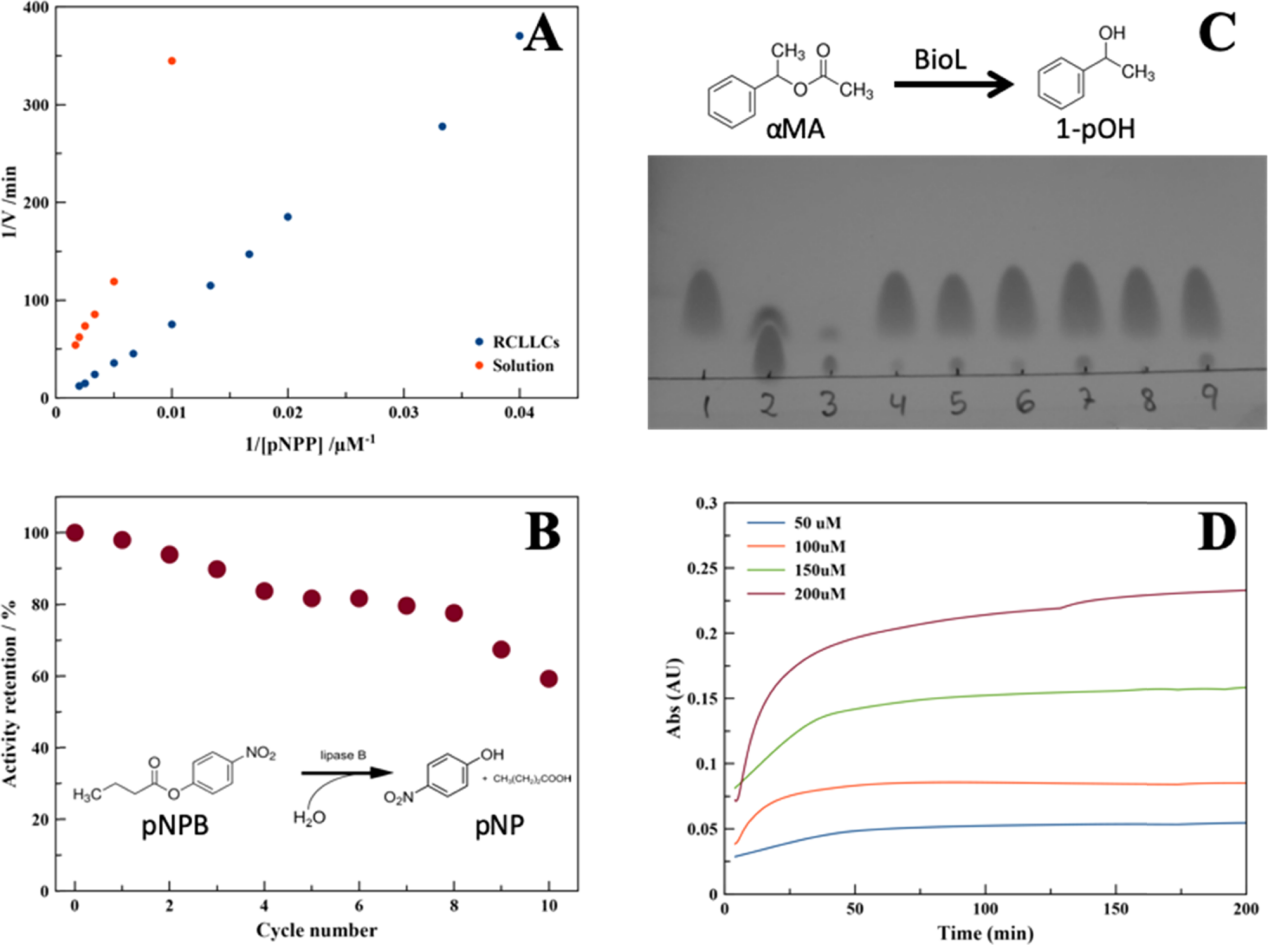
(A) Comparison of the activity of BioL in a solution (red, 50 μg·mL^–1^) and as RCLLCs (blue, 50 μg RCLLCs·mL^–1^), using pNPB as a substrate (25–600 μM). (B) Activity of RCLLCs (1.0 mg) vs the number of reaction cycles. (C) Hydrolysis of αMA using BioL in a solution (0.8 mg·mL^–1^) and in a crystalline state (8.3 mg·mL^–1^) showed in a thin-layer chromatography (TLC) plate: lane 1, α-methylbenzyl acetate (αMA); lanes 2 and 3, 1-phenylethanol (1-pOH) at two concentrations (pure and 1/10 dilution); lanes 4, 6, and 8, hydrolysis of αMA using soluble BioL in heptane at 40, 50, and 60 °C, respectively; and lanes 5, 7, and 9, hydrolysis of αMA using RCLLCs in heptane at 40, 50, and 60 °C, respectively. (D) Continuous production of pNP produced by the RCLLC packed column at different initial substrate concentrations (50–200 μM) under a continuous flow of 1.0 mL·min^–1^.

The activity of RCLLCs was assayed using an effective crystal concentration of 50 μg·mL^–1^, with substrate concentrations ranging from 25 to 600 μM. Under these conditions, RCLLCs showed an effective activity of approximately 1 order of magnitude lower than the activity of the free BioL in a solution (Figure 5A). Although this difference should be lower, since the composition of the RCLLCs also includes other components such as the agarose used for crystallization, this result was expected since the diffusion of the substrate and the product in/out the crystals limits the apparent catalytic performance.^90^ This apparent drawback is clearly overcome when the solid form of the enzyme is reused in a number of cycles. Figure 5B shows the activity retention as a function of the number of cycles. The apparently lower BioL activity was due to the loss of crystals during the washing procedure between cycles. Normalization of the activity considering the mass of the final RCLLCs (0.3 mg vs the initial 0.5 mg) indicated retention of almost 100% activity after 10 reuses.

We also tested the compatibility of the RCLLCs with an organic solvent. The hydrolysis of α-methylbenzyl acetate in heptane to produce 1-phenylethanol was analyzed using TLC (Figure S6). In this case, and following the previous observation, we used 10 times more enzyme in the crystalline form than in the solution to get a similar level of hydrolysis (Figure S6). We also used this reaction to compare the thermal stability of RCLECs vs BioL in organic media. As shown in Figure 5C, while dissolved BioL is completely inactive after 24 h at 60 °C, the RCLLC solid form remains fully active. Thus, immobilization of BioL as RCLLCs also resulted in an improved stability, as has been shown for other lipases after immobilization in solid supports.^91,92^

The best way to exploit the retention of the activity avoiding the loss of crystals is their use in a continuous mode provided by a preparative chromatographic column. This approach has already been tested with supported lipase, and in one case, with the autosupported CLEA prepared from lipase from *C. rugosa*, but in the latter case, the specific activity decayed with the number of cycles.^93^ Although there have been early studies on the use of packed-bed columns using CLECs of alcohol dehydrogenase,^94^ only in the case of laccase the use of CLEC in a packed-bed configuration has been reported and fully characterized. The laccase-CLEC column was effectively employed in a continuous flow to convert pyrogallol to purpurogallin with activity retention of 76.28% and with a catalyst to product ratio of 1:2241. Since this reaction was fast and the separation of the product reasonably easily achieved, with no byproducts or hazardous chemical generation, the overall process was considered very economical.^95^

In our case, dried RCLLC crystals were mixed with glass beds to pack the bed column, as described in Materials and Methods section. A single column of 10.0 cm length (0.4 cm diameter) was used to run all described experiments. Figure 5D shows the continuous production of pNP (absorbance at 400 nm) as a function of time produced by the RCLLC packed column at different initial substrate concentrations (50–200 μM) under a continuous flow of 0.25 mL·min^–1^. We tested several flow rates of the substrate, 0.25, 0.5, and 1.0 mL·min^–1^at different initial substrates concentration and found not much difference (Figure S7). In these conditions, the maximum conversion rate (substrate/product) was achieved at the lowest substrate concentration and the fastest flow rate. On the other hand, at the highest substrate concentration (200 μM), the lowest flow rate produced more product as expected due to the longer reaction time (Figure S7). To determine how much substrate could be converted, we operated the RCLLC packed-bed column in a close recirculating system of 600 mL total volume at a fixed flow rate of 0.25 mL·min^–1^. We reached 65% conversion operation after 16.7 h when the initial substrate concentration was 200 μM and 40% conversion in 13.3 h if starting from 50 μM substrate (Figure S8).

We did not extenuate the column even though all of the assays were carried out in a continuous flow with the substrate at different concentrations and including the cleaning steps, demonstrating the robustness of the system.

## Conclusions

In this work, we have successfully produced agarose-reinforced lipase crystals, which were cross-linked in situ to obtain RCLLCs starting from a commercial detergent additive. Using the batch method in agarose media, we scaled-up the production of RCLLCs to 500 mL, facilitating crystals’ extraction in a thermal batch that could be done automatically. RCLLCs were lyophilized and their enzymatic activities were characterized both in water and heptane. As it has already been shown for other CLECs, RCLLCs presented higher thermal stability and tolerance to an organic solvent than the free protein in a solution. Lyophilized RCLLCs were used to pack a chromatographic column that was able to work under continuous flow conditions for an extended period of time. The conversion of pNPB to pNP was achieved and spectrophotometrically followed at different flow rates. More than 10 L of the substrate was circulated through the column and a similar volume of cleaning buffer between reactions, demonstrating the robustness of the system.

The method presented here is easily applicable to any other enzyme having crystallization conditions compatible with agarose gelation. For temperature-sensitive enzymes (either due to low thermal stability or crystal solubility), low gelling temperature agaroses can be used.

## Supporting Information Available

Further details on crystallization optimization (Figures S1–S3), crystal size determination (Figure S4), the calibration curve of *p*NP vs absorbance (Figure S5), and the enzymatic activity (Figures S6–S8) (PDF)

## Abbreviations

CLPC: cross-linked protein crystals
CLEC: cross-linked enzyme crystals
CLEA: cross-linked enzyme aggregates
RCLEC: reinforced cross-linked enzyme crystals
RCLLCs: reinforced cross-linked lipase crystals
*p*NPB: *p*-nitrophenyl butyrate
*p*NP: *p*-nitrophenol
BioL: biolipase (commercial lipase)
TLC: thin-layer chromatography
TLL: lipase from *Thermomyces lanuginosus*
αMA: α-methylbenzyl acetate
