# Supplementary material for: Production of Cross-Linked Lipase Crystals at a Preparative Scale
Source: Cryst Growth Des. 2021 Feb 17;21(3):1698–707. doi: 10.1021/acs.cgd.0c01608 (PMC8479976; doi:10.1021/acs.cgd.0c01608)
Supplement: Supplementary file 1 — cg0c01608_si_001.pdf [file cg0c01608_si_001.pdf]

ELECTRONIC SUPPORTING INFORMATION

Production of cross-linked lipase crystals at preparative scale

Raquel Fernández-Penas[1], Cristóbal Verdugo-Escamilla[1], Sergio Martínez-Rodríguez[1,2], José A. Gavira[1]*

[1] Laboratorio de Estudios Cristalográficos, Instituto Andaluz de Ciencias de la Tierra (Consejo Superior de Investigaciones Científicas-Universidad de Granada), Avenida de las Palmeras 4, 18100 Armilla, Granada, Spain. [2] Dpto. Bioquímica y Biología Molecular III e Inmunología (Universidad de Granada). Avda. de la Investigación 11, 18071, Granada, Spain.

*Correspondence to: jgavira@iact.ugr-csic.es

Figures and Tables

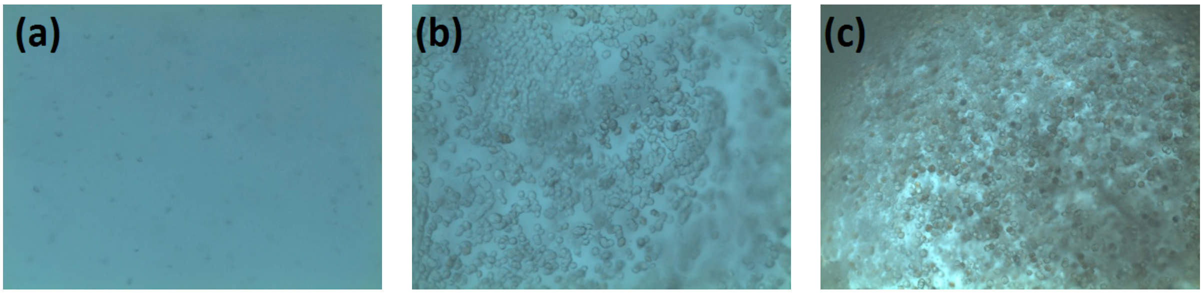

Figure S1. Optimization of the crystallization conditions of lipase (30 mg/ml). The amount of precipitant K/NaH_2PO_4 was 50 (a), 100 (b) and 150 mM (c), respectively. Pictures were taken with the same magnification.

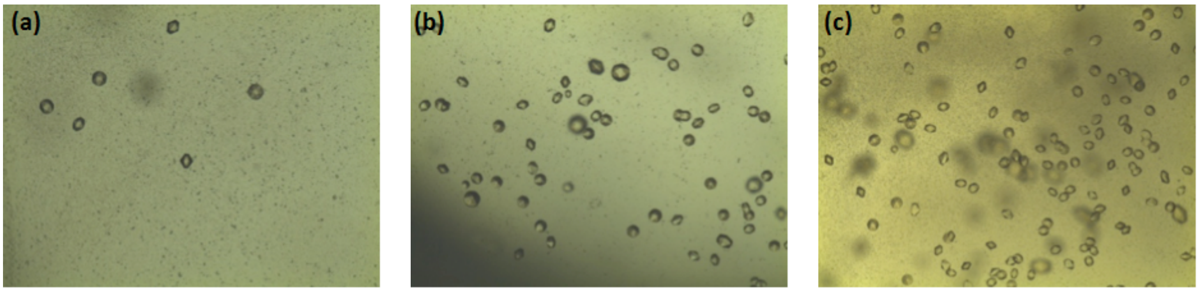

Figure S2. Optimization of the crystallization conditions of lipase by keeping the precipitant concentration at 100 mM of K/NaH_2PO_4 and changing the protein concentration, 5 (a), 10 (b) and 20 mg/ml of lipase. Pictures were taken with the same magnification.

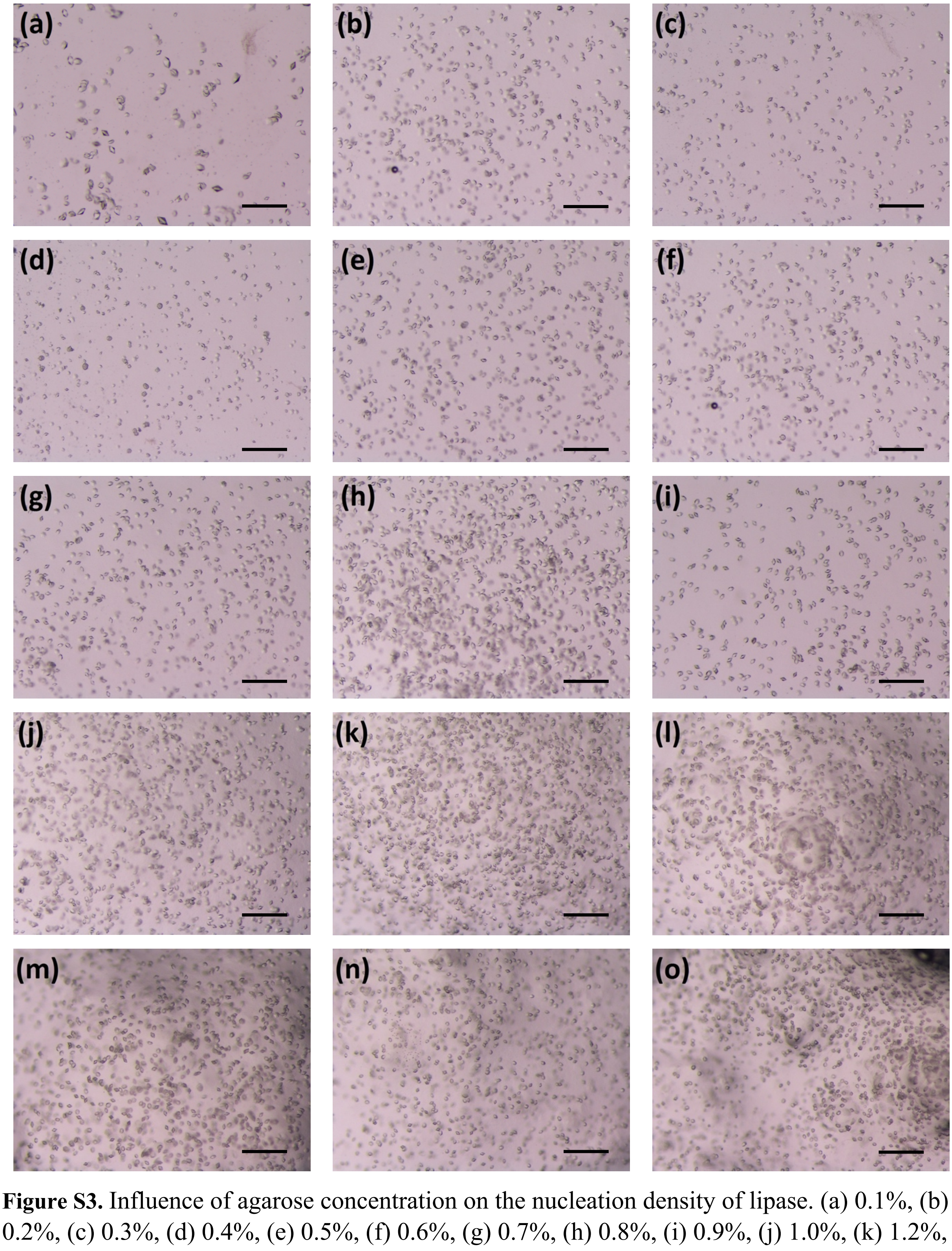

Figure S3. Influence of agarose concentration on the nucleation density of lipase. (a) 0.1%, (b) 0.2%, (c) 0.3%, (d) 0.4%, (e) 0.5%, (f) 0.6%, (g) 0.7%, (h) 0.8%, (i) 0.9%, (j) 1.0%, (k) 1.2%, (l) 1.4%, (m) 1.6%, (n) 1.8% and (o) 2.0% w/v, respectively. All images were taken with the same magnification. Scale bars are for 500 μm.

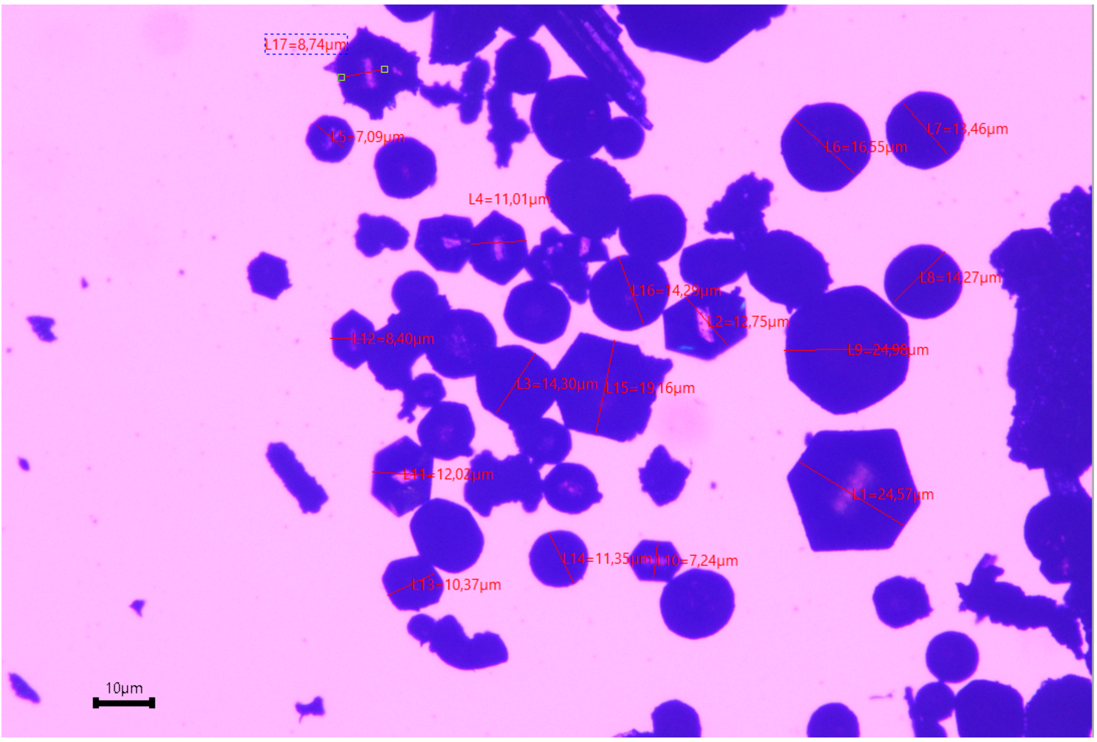

Figure S4. Example of crystals size determination using the ImageJ software.

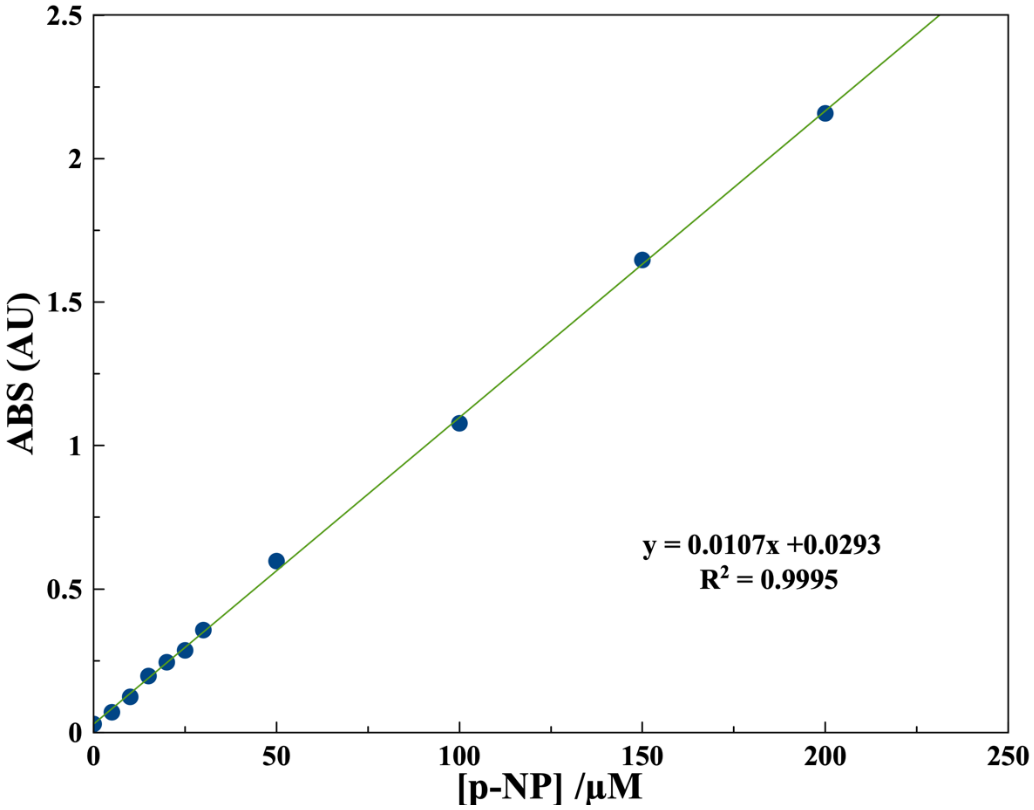

Figure S5. Calibration curve correlating the absorbance and concentration of p-NP. p-NP solutions were prepared from the pure commercial product.

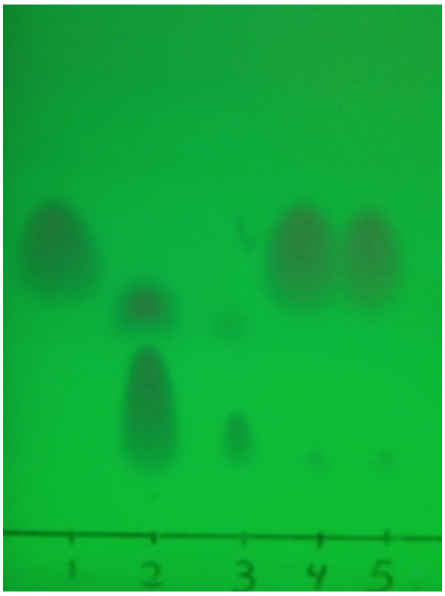

Figure S6. Thin layer chromatography analysis of reaction products of BioL in solution (L4) and as RCLLCs (L5) in heptane at 40°C/24 hours, using α-Methylbenzyl acetate as substrate (L1). The product, 1-phenylethanol, was run in L2 and L3 (1/10 dilution) as references.

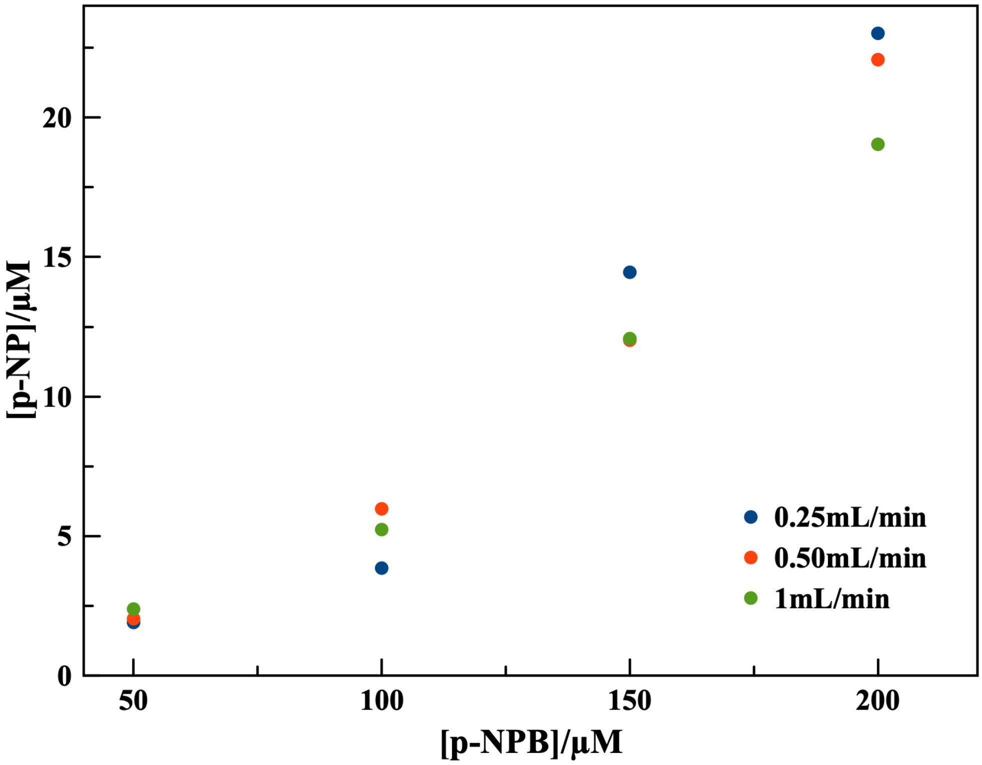

Figure S7. Amount of p-NP produced by the RCLLCs packed column as a function of the initial substrate concentration circulating at 0.25, 0.5 and 1.0 mL/min.

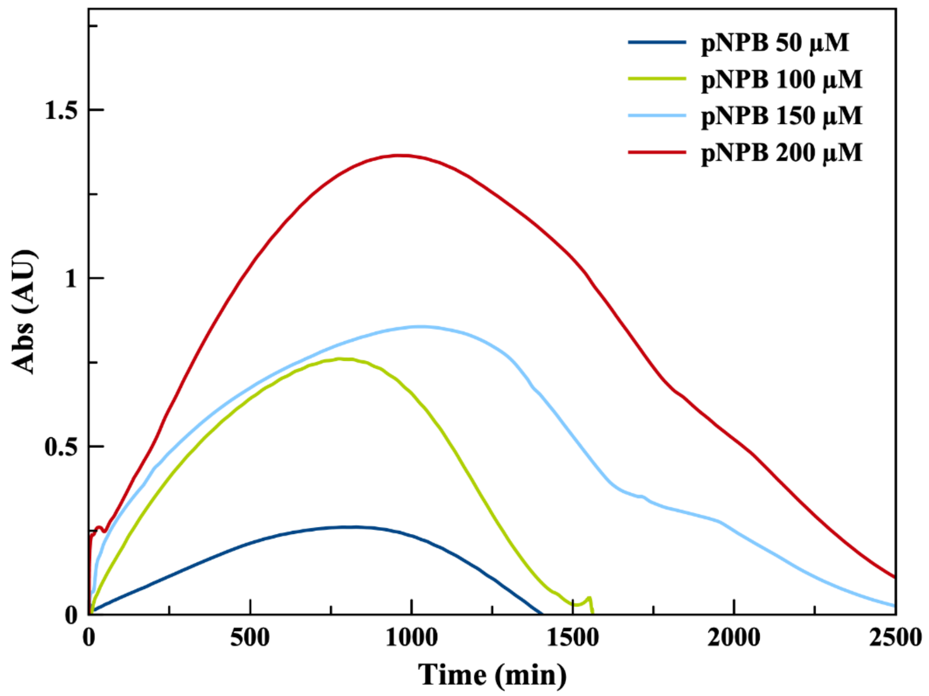

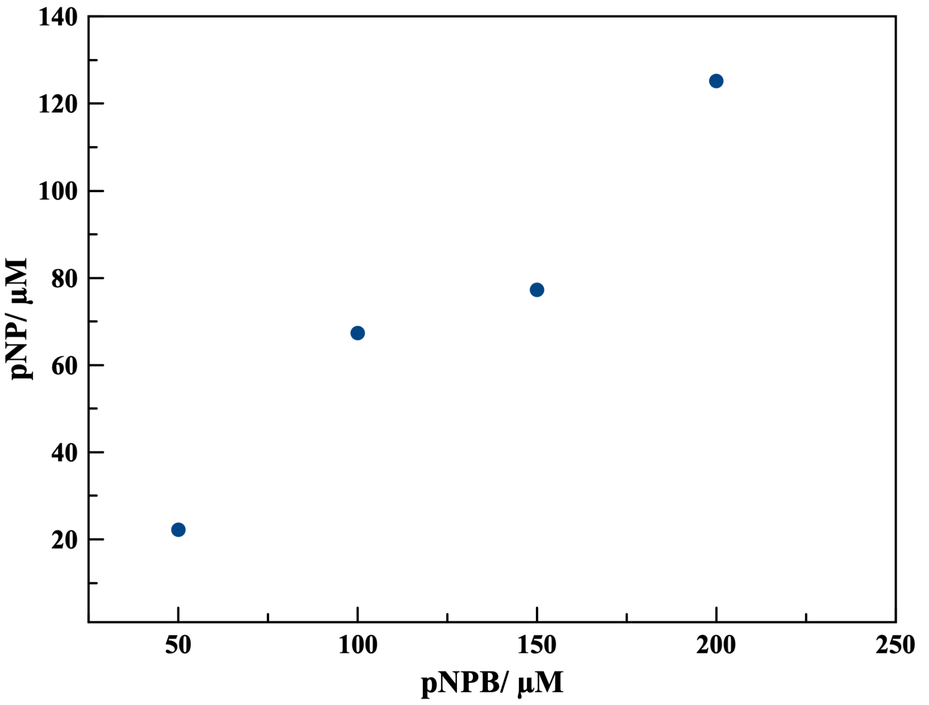

Figure S8 A. Determination of the maximum of production of p-NP (absorbance at 400 nm) produced by the RCLLCs packed column at different initial substrate concentrations (50 to 200 µM) under a continuous flow of 0.25 mL/min in a close re-circulating system as a function of time. The decay of the signal is due to the auto-hydrolysis of the substrate in the blank cell. B. The maximum of conversion is represented versus the initial substrate concentration.
